# Associations between area- and individual-level community belonging and self-rated health

**DOI:** 10.1093/eurpub/ckac129.308

**Published:** 2022-10-25

**Authors:** S Mah, M Brown, R Colley, R Rosella, C Sanmartin, G Schellenberg

**Affiliations:** 1 Dalla Lana School of Public Health, University of Toronto, Toronto, Canada; 2 Statistics Canada, Ottawa, Canada

## Abstract

**Background:**

Previous studies point to the importance of individuals’ sense of community belonging to multiple measures of health and well-being. However, the extent to which collective sense of belonging within neighbourhoods might influence individual health has not been characterized. The objectives of this study are to describe variations in self-rated health by a novel area-level measure of community belonging and determine the impact of including these measures in models of individual-level community belonging and self-rated health.

**Methods:**

We conducted a cross-sectional study of respondents of the 2020 Canadian Community Health Survey (CCHS) living in census tracts. These data were merged with novel small area estimates of community belonging derived by Statistics Canada from the CCHS 2016-2019. Multinomial logistic regression models were used to analyse associations of individual- and area-level community belonging, and self-rated health. We adjusted for sex, age, recent immigrant status, visible minority status, province, marital status, presence of children in the household, smoking status, presence of chronic conditions, income, and employment status. All results were generated using survey weights and bootstraps representing a subpopulation of 21 million people.

**Results:**

A greater proportion of CCHS respondents living in neighbourhoods with the strongest collective sense of community belonging reported being in good, very good, or excellent health. Models indicate that living in a neighbourhood with low collective sense of community belonging is associated with higher odds of reporting being in poor or fair health (OR = 1.44, 95% CI 1.15-1.81) even after adjusting for other factors, including individual-level sense of community belonging, which also remained independently associated with self-rated health.

**Conclusions:**

Both area- and individual-level sense of community belonging are independently associated with self-rated health.

**Key messages:**

• The collective sense of belonging within neighbourhoods may influence health outcomes.

• Measures of area-level and individual-level community belonging may capture different aspects of social connections with respect to health.

